# Comparing transplant outcomes in ALL patients after myeloablative conditioning in mismatch-related or unrelated donor settings

**DOI:** 10.1038/s41409-024-02378-0

**Published:** 2024-08-15

**Authors:** Salman Otoukesh, Dongyun Yang, Sally Mokhtari, Hoda Pourhassan, Vaibhav Agrawal, Shukaib Arslan, Idoroenyi Amanam, Brian Ball, Paul Koller, Amandeep Salhotra, Karamjeet Sandhu, Ahmed Aribi, Andrew Artz, Ibrahim Aldoss, Vinod Pullarkat, Haris Ali, Amanda Blackmon, Pamela Becker, Peter Curtin, Forrest Stewart, Eileen Smith, Anthony Stein, Guido Marcucci, Stephen J. Forman, Ryotaro Nakamura, Monzr M. Al Malki

**Affiliations:** 1 Department of Hematologic Malignancies and Translational Science, City of Hope National Medical Center, Duarte, CA USA; 2 Department of Computational and Quantitative Medicine, City of Hope National Medical Center, Duarte, CA USA; 3 Department of Clinical and Translational Project Development, City of Hope National Medical Center, Duarte, CA USA

**Keywords:** Translational research, Cancer therapy, Acute lymphocytic leukaemia

## Abstract

The optimal myeloablative conditioning regimen for ALL patients undergoing hematopoietic cell transplant (HCT) with an alternative donor is unknown. We analyzed HCT outcomes ALL patients (*n* = 269) who underwent HCT at our center from 2010 to 2020 in complete remission (CR) after FTBI-etoposide and CNI-based GvHD prophylaxis for matched donor HCT (ETOP-package; *n* = 196) or FTBI-Fludarabine and post-transplant cyclophosphamide (PTCy)-based prophylaxis for HLA- mismatched (related or unrelated) donors (FLU-package; *n* = 64). Patients in FLU-package showed a significant delay in engraftment (*p* < 0.001) and lower cumulative incidence (CI) of any and extensive chronic GVHD (*p* = 0.009 and 0.001, respectively). At the median follow up of 4.6 years (range 1–12 years); non-relapse mortality, overall or leukemia-free survival and GVHD-free/relapse-free survival were not significantly impacted by the choice of conditioning. However, in patients at CR2 or with measurable residual disease (MRD+), there was a trend towards higher relapse after FLU-package (*p* = 0.08 and *p* = 0.07, respectively), while patients at CR1 regardless of MRD status had similar outcomes despite the package/donor type (*p* = 0.9 and 0.7, respectively). Our data suggests that FLU-package for alternative donors offers comparable outcomes to ETOP-package for matched donor HCT to treat ALL. Disease status and depth of remission at HCT were independent predictors for better outcomes.

## Introduction

Allogeneic hematopoietic cell transplantation (HCT) represents the first curative therapy for adults with acute lymphoblastic leukemia (ALL) [1–3]. In leukemia, HCT impacts the underlying malignancy directly by eliminating cancer cells through pre-transplant conditioning regimens and indirectly through post-transplant graft-versus-leukemia (GVL) effect [4]. The cytotoxic effect of preparative regimen is known to have an important role in treatment outcomes, and Fractionated Total Body Irradiation (FTBI) in the range of 12–13.2 Gy is considered the main backbone for conditioning in fit patients who are eligible to receive myeloablative conditioning [5–7].

The first successful myeloablative HCT of FTBI (12 Gy) with Cyclophosphamide (Cy) was done in 1979 [8]. Since then, other chemotherapy agents (i.e., Cytarabine, Melphalan, and Busulfan) have been combined with FTBI as pre-HCT conditioning to treat different hematologic malignancies, with no evidence of superiority in outcome when compared to TBI/Cy [9–11]. FTBI (13.2 Gy) and Etoposide (60 mg/kg) as conditioning regimen for matched related donor HCT to treat patients with ALL was first described by City of Hope and Stanford in 2006 [7]. This regimen was shown to be effective in reducing the risk of relapse, treatment failure and overall mortality in patients with ALL in their first or second complete remission (CR). This report was then validated and adopted as standard of care by the International Medical Research Council UKALL12/Eastern Cooperative Oncology Group study with 5-year disease-free survival (DFS) of 45% and 100-day transplant-related mortality of 21% [12, 13].

In the last decade, alternative donors including mismatched unrelated (MMUD) and haploidentical (HAPLO), have provided a donor for almost every patient [14, 15], with comparable transplant outcomes to fully matched donors [16–20]. Administration of post-transplant cyclophosphamide (PTCy), has significantly reduced rates of GVHD and improved outcomes in patients undergoing Haplo and MMUD HCT [19, 20]. In a large, multi-center retrospective analysis, Al Malki et al., reported similar HCT outcomes of acute and chronic GVHD and overall survival (OS) among patients with ALL undergoing HAPLO HCT with PTCy-based GVHD prophylaxis or matched unrelated donor (MUD) HCT with conventional GVHD prophylaxis. Myeloablative regimens were used in 74% of both groups [19]. In the largest reported single-center experience, Agrawal et al. reported the superiority of HCT outcomes in PTCy-based GVHD prophylaxis compared to conventional GVHD prophylaxis regimens [5-year OS: *P* = 0.045, 5-year DFS: *P* = 0.042, and cumulative incidence (CI) of chronic GVHD: *P* < 0.001] in the setting of MMUD [21]. In a recent publication from the Acute Leukemia Working Party of the European Society for Blood and Marrow Transplantation, PTCy was associated with better leukemia-free survival (LFS) compared to rabbit anti-thymocyte globulin (ATG), in patients with ALL in CR1 who underwent HCT from matched unrelated donors [22]. TBI-based conditioning was delivered in 56% of patients in the PTCy group and 63% of patients in the ATG group.

Despite the increase in the use of alternative donor HCT, the optimal FTBI-based conditioning regimen in patients with ALL undergoing mismatched related or unrelated HCT is not yet established. In this study, we retrospectively analyzed outcomes of patients with ALL who underwent FTBI-based myeloablative HCT, based on donor status (matched unrelated or mismatched related/unrelated) and GVHD prophylaxis.

## Subjects and methods

### Study design and data collection

This was a retrospective, single-center study with a total of 269 patients with ALL in their first or second+ CR (CR1, CR2+) who underwent their first HCT from either a matched donor (MD: sibling or unrelated of 8/8 allele-matched), or mismatched related (HAPLO) or unrelated (MMUD) between January 2010 and December 2020 at City of Hope National Medical Center.

### Ethics approval and consent to participate

This study was reviewed and approved by the City of Hope Institutional Review Board and was performed in compliance with the Declaration of Helsinki. Only patients who signed an informed consent prior to participation were included.

### Treatment plan

Patients with a matched donor received the “ETOP package”, which consisted of FTBI at 13.2 Gy delivered at 1.2 Gy per fraction three times per day on Days -7 to -4 pre-HCT), and Etoposide (ETOP, 60 mg/kg on Day -3 pre-HCT) as conditioning regimen; and tacrolimus (Tacro; 0.02 mg/kg IV on Day -2 pre-HCT) and sirolimus (Siro; 12 mg PO on Day -2 followed by 4 mg PO on Day -1 before HCT) as GVHD prophylaxis. Patients with a mismatched related or unrelated donor received the “FLU package”, which consisted of FTBI at 1200 Gy delivered at 1.5 Gy per fraction twice a day on Days -4 to -1 pre-HCT, and fludarabine (FLU; 30 mg/m^2^ IV on Days -7 to -5 pre-HCT) as conditioning regimen and PTCy (50 mg/kg IV D + 3 and D + 4 post-HCT), Mycophenolate Mofetil (MMF, 1000 g IV on Day +5 post-HCT), and Tacro (1 mg IV on Day +5 post-HCT) as GVHD prophylaxis. TBI was delivered using established techniques per previously published guidelines by the International Lymphoma Radiation Oncology Group.. Patients were treated in the standing position at an extended distance [23]. Lung shielding was utilized for all fractions using 50% partial transmission blocks. The dose-rate was 10–15 cGy per minute. All patients received peripheral blood stem cells as graft source.

### Measurable residual disease (MRD) assessment

MRD was assessed within 30 days from HCT either by 12-color flow cytometry in patients with Philadelphia chromosome-negative ALL, as well as for T cell receptor chain rearrangement in T-ALL, or by PCR BCR/ABL1 in patients with Philadelphia chromosome-positive ALL. Patient was considered MRD positive vs. negative based on the assessment of one of these tests in the correct setting.

### Endpoints and definitions

The primary endpoint was LFS defined as time from date of HCT to first observation of disease relapse/progression or death, whichever came first. LFS was censored at the last follow-up if patients who remained alive and free of disease. Cause of death (COD) was attributed to (1) relapse, (2) GVHD (active GVHD or if patient was taking 20 mg prednisone or its equivalent with no relapse), (3) infection (with no relapse or active GVHD and documented infection mortality), and (4) organ failure not related to relapse/GVHD/infection. Relapse was defined as clinical or hematological leukemia recurrence. Secondary endpoints included neutrophil and platelet engraftments, acute GVHD, chronic GVHD, non-relapse mortality (NRM), relapse, OS, and GVHD-free and relapse-free survival (GRFS). Neutrophil engraftment was defined as the first three consecutive days of an absolute neutrophil count (ANC) of >0.5 × 10^9^/L. Platelet engraftment was defined as platelet count >20 × 10^9^/L for three consecutive days with no transfusion in the previous week. Grades 2–4 and 3–4 acute GVHD were defined by the Glucksberg scale [24] and chronic GVHD was defined as limited or extensive chronic GVHD according to the Seattle criteria [25]. Cumulative incidence of relapse was defined as time from transplant to the first observation of disease relapse/progression. Cumulative incidence of NRM was defined as time from transplant to the date of death if disease relapse/progression was not observed before death. Relapse and NRM were competing risks events to each other. Relapse and NRM were censored at the last follow-up if patients were alive and free of disease. Relapse and NRM were considered as competing risk events for engraftment and GVHD. OS was defined as the time from transplant to date of death or censored at the last follow-up if patients were last known to be alive. GRFS was defined as the time from transplant to the date of grade 3–4 acute GVHD, moderate or severe chronic GVHD based on NIH criteria [26], relapse, or death, whichever came first. GRFS was censored at the last follow-up if patients were alive and free of these events.

### Statistical considerations

Descriptive statistics were used to compare the baseline patient, disease, and transplant characteristics by the myeloablative conditioning regimens. Two-sample Wilcoxon tests were used to compare continuous variables and chi-square tests were used to compare categorical variables by the myeloablative conditioning regimens. Kaplan-Meier curves and log-rank tests were used in the univariate analysis (UVA) and Cox regression models were used in the multivariable analysis (MVA) of OS and LFS. Cumulative incidence curves and Gray’s test were used in the UVA and Fine and Gray regression models were used in the MVA of relapse, NRM, engraftment, and GVHD.

Stepwise regression strategies were used to choose the baseline variables that were included in the multivariable regression models. Variables that were significantly associated with an outcome at 0.1 level were kept in the final multivariable model.

All p values were two-sided at a significance level of 0.05. SAS 9.4 (SAS Institute, Cary, NC) was used to conduct the analyses and generate curves.

## Results

### Patient and HCT characteristics

Table 1 shows detailed patients, diseases, and transplant characteristics. Briefly, a total of 269 patients with ALL underwent one of the two HCT (conditioning and GVHD) packages, assigned based on the donor matching status, including ETOP (*n* = 205) or FLU (*n* = 64), with a median follow-up duration of 4.6 years (range: 1–12 years). The median age at the time of HCT for all patients was 37 years (range: 9-68). More than half of the patients were male (59.5%), and Hispanic (59.5%). Majority of the patients were diagnosed with B-cell ALL (86.6%) and the rest had T-cell ALL(13.4%). Most of the patients (79.2%) underwent HCT in CR1. High cytogenetic risk was reported in 203 patients (75.5%). Philadelphia(Ph) -negative status was reported in 167 patients (62.1%), of whom 29 patients had Philadelphia-like (Ph-like) ALL, and 102 patients (37.9%) had Ph-positive disease. Post-HCT Tyrosine Kinase Inhibitors (TKI) maintenance therapy was administered in 61% of patients with Ph-positive ALL (*n* = 62/102), including 58% of Ph-Positive patients in the ETOP-package and 68% of Ph-positive patients in the FLU-package. Use of post-HCT TKI maintenance therapy was not statistical difference in ETOP-package compared to FLU-package (58 vs 68%, *P* = 0.4).

**Table 1:** Patient and transplant characteristics.

	FTBI/ETOP (*N* = 205)	FTBI/FLU (*N* = 64)	Total (*N* = 269)	*p* value
Age at HSCT, years				0.55
Median (Range)	37 (19-59)	33 (9-68)	37 (9-68)	
Recipient sex				0.14
Male	127 (62%)	33 (51.6%)	160 (59.5%)	
Female	78 (38%)	31 (48.4%)	109 (40.5%)	
Race/ethnicity				0.0095
White	57 (27.8%)	9 (14.1%)	66 (24.5%)	
African American	2 (1%)	0 (0%)	2 (0.7%)	
Asian	28 (13.7%)	4 (6.3%)	32 (11.9%)	
Hispanic	113 (55.1%)	47 (73.4%)	160 (59.5%)	
Native	4 (2%)	1 (1.6%)	5 (1.9%)	
Not reported	1 (0.5%)	3 (4.7%)	4 (1.5%)	
Karnofsky performance status %				0.19
80–100	189 (92.2%)	62 (96.9%)	251 (93.3%)	
≤70	16 (7.8%)	2 (3.1%)	18 (6.7%)	
HCT comorbidity index				0.0002
0	77 (37.6%)	7 (10.9%)	84 (31.2%)	
1–2	70 (34.1%)	28 (43.8%)	98 (36.4%)	
≥3	58 (28.3%)	29 (45.3%)	87 (32.3%)	
Disease type				0.13
B-ALL	174 (84.9%)	59 (92.2%)	233 (86.6%)	
T-ALL	31 (15.1%)	5 (7.8%)	36 (13.4%)	
Disease status				0.045
CR1	168 (82%)	45 (70.3%)	213 (79.2%)	
CR2+	37 (18%)	19 (29.7%)	56 (20.8%)	
MRD status				0.66
MRD+	42 (31.3%)	16 (28.1%)	58 (21.6%)	
MRD-	92 (68.7%)	41 (71.9%)	133 (49.4%)	
NA	71	7	78 (29%)	
Cytogenetic risk				0.92
Standard	50 (24.4%)	16 (25%)	66 (24.5%)	
High	155 (75.6%)	48 (75%)	203 (75.5%)	
Philadelphia status				0.82
Ph+	77 (37.6%)	25 (39.1%)	102 (37.9%)	
Ph-	107 (52.2%)	31 (48.4%)	138 (51.3%)	
Ph-like	21 (10.2%)	8 (12.5%)	29 (10.8%)	
Female donor to male recipient				0.18
Yes	52 (25.4%)	11 (17.2%)	63 (23.4%)	
No	153 (74.6%)	53 (82.8%)	206 (76.6%)	
Donor age				0.20
Median (Range)	33 (11-62)	31 (14-65)	32 (11-65)	
Interquartile range	27, 43	23, 42	26, 43	
Donor Type				<0.0001
MUD	192 (93.7%)	4 (6.3%)	196 (72.9%)	
MMUD	13 (6.3%)	13 (20.3%)	26 (9.7%)	
Haplo	0 (0%)	47 (73.4%)	47 (17.5%)	
ABO blood group compatibility				0.31
ABO compatible	134 (65.4%)	42 (65.6%)	176 (65.4%)	
Minor mismatch (donor is O)	27 (13.2%)	5 (7.8%)	32 (11.9%)	
Major mismatch (Recipient is O)	31 (15.1%)	9 (14.1%)	40 (14.9%)	
Bidirectional (None are O)	13 (6.3%)	8 (12.5%)	21 (7.8%)	
Donor/Recipient CMV serostatus				0.34
D-/R-	19 (9.3%)	6 (9.4%)	25 (9.3%)	
D-/R+	53 (25.9%)	11 (17.2%)	64 (23.8%)	
D+/R-	10 (4.9%)	6 (9.4%)	16 (5.9%)	
D+/R+	123 (60%)	41 (64.1%)	164 (61%)	
HCT period				
2010-2014	83 (40.5%)	0 (0%)	83 (30.9%)	
2015-2020	122 (59.5%)	64 (100%)	186 (69.1%)	
GVHD prophylaxis				
Cyclophosphamide/Tacrolimus/MMF	0 (0%)	64 (100%)	64 (23.8%)	
Tacrolimus/Sirolimus	205 (100%)	0 (0%)	205 (76.2%)	

Pre-HCT MRD status was available in 191 patients of whom 58 patients (21.6%) had MRD+ status at the time of HCT. MRD was assessed by 12-color flow cytometry in 61% (*n* = 102/167) of patients with Ph-negative (including Ph-like) and 87% (*n* = 89/102) of patients with Ph-positive ALL. In patients who had MRD assessed by PCR-BCR/ABL1, 30% (*n* = 58/191) were MRD positive with no significant difference between ETOP and FLU (31% vs 28%, *p* = NS).

Both groups had comparable characteristics, but ETOP had more patients in CR1 (82% vs. 70%, *p* = 0.045) and a better HCT comorbidity index (HCT-CI of 0 was 37% vs. 11%, *p* = 0.0002) compared to FLU.

### Engraftment

**Fig. 1:**
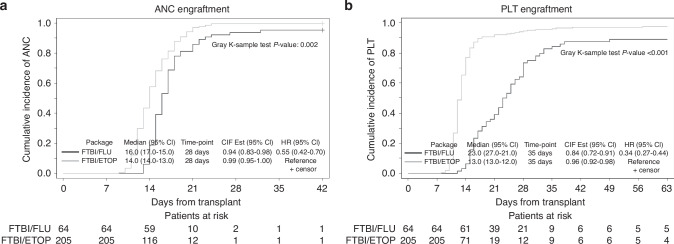
Engraftment outcomes in patients with ALL receiving FTBI/FLU or FTBI/ETOP package. **a** Neutrophil engraftment, **b** Platelet engraftment.

Median time to neutrophil engraftment was 2 days shorter in the ETOP (14 days compared to 16 days in the FLU group). Neutrophil engraftment by day +28 was detected in 99% of patients with ETOP and 94% of patients with FLU (*p* = 0.002). (Fig. 1a)

Median time to platelet recovery in the ETOP group was 13 days compared to 23 days in the FLU group. At 35 days post-HCT, platelet engraftment was achieved in 96% of patients in the ETOP and 85% of patients in the FLU groups. (Fig. 1b)

These results were confirmed by multivariate analysis (MVA), with a significant delay in day-28 neutrophil recovery (HR = 0.55, 95% CI: 0.43–0.70, *p* < 0.001) and day-42 platelet engraftment (HR = 0.29, 95% CI: 0.22–0.39, *p* < 0.001) in FLU compared to ETOP (Table 2).

**Table 2:** Multivariate analysis of engraftment, relapse, and survival and GVHD outcomes.

			*Neutrophil Engraftment*		*Platelet Engraftment*	
		*N*	*Adjusted HR (95%CI)*^*a*^	*FG test P*^*a*^	*Adjusted HR (95%CI)*^*a*^	*FG test P*^*a*^
Disease status	CR1	213	Reference	0.67	Reference	**<0.001**
	CR2+	56	0.94 (0.71,1.24)		0.59 (0.43,0.80)	
HCT period	2010–14	83	Reference	0.37	Reference	**0.018**
	2015–20	186	0.88 (0.67,1.16)		1.51 (1.07,2.11)	
FTBI+	VP-16	205	Reference	**<0.001**	Reference	**<0.001**
	FLU	64	0.55 (0.43,0.70)		0.29 (0.22,0.39)	

			*Relapse*		*NRM*	
		*N*	*Adjusted HR (95%CI)*^*b*^	*FG test P*^*b*^	*Adjusted HR (95%CI)*^*b*^	*FG test P*^*b*^
Ph status	Ph+	102	Reference	**0.023**	Reference	0.67
	Ph-	138	1.66 (0.75,3.65)		0.84 (0.48,1.46)	
	Ph-like	29	3.72 (1.44,9.58)		0.67 (0.25,1.77)	
Disease status	CR1	213	Reference	0.20	Reference	**0.018**
	CR2+	56	1.59 (0.78,3.23)		1.99 (1.12,3.50)	
F Donor to M	No	206	Reference	0.093	Reference	**0.010**
	Yes	63	0.45 (0.18,1.14)		2.05 (1.19,3.53)	

			*Overall Survival*		*LFS*	
		*N*	*Adjusted HR (95%CI)*^*c*^	*Wald test P*^*c*^	*Adjusted HR (95%CI)*^*c*^	*Wald test P*^*c*^
Disease status	CR1	213	Reference	**<0.001**	Reference	**0.001**
	CR2+	56	2.34 (1.49,3.67)		2.08 (1.34,3.21)	

			*Grade 2–4 acute GVHD*		*Grade 3–4 acute GVHD*	
		*N*	*Adjusted HR (95%CI)*	*FG test P*^*d*^	*Adjusted HR (95%CI)*	*FG test P*^*d*^
Sex	Male	160	Reference	**0.024**	Reference	**0.041**
	Female	109	0.65 (0.45,0.95)		0.50 (0.25,0.97)	
Donor	Matched	196	Reference	**0.010**	Reference	0.63
	MMUD	26	2.17 (1.30,3.63)		1.33 (0.49,3.61)	
	HAPLO	47	0.94 (0.58,1.53)		0.73 (0.30,1.74)	

			*Any chronic GVHD*		*Extensive chronic GVHD*	
		*N*	*Adjusted HR (95%CI)*	*FG test P*^*e*^	*Adjusted HR (95%CI)*	*FG test P*^*e*^
Donor	Matched	196	Reference	0.089	Reference	0.021
	MMUD	26	0.70 (0.39,1.25)		0.58 (0.30,1.12)	
	HAPLO	47	0.62 (0.39,1.00)		0.52 (0.31,0.89)	
F donor to M	No	206	Reference	0.013	Reference	0.007
	Yes	63	1.49 (1.09,2.05)		1.60 (1.14,2.24)	
HCT period	2010–2014	83	Reference	0.013	Reference	0.011
	2015–2020	186	0.67 (0.48,0.92)		0.65 (0.46,0.91)	
FTBI+	ETOP	205	Reference	0.009^f^	Reference	0.001^f^
	FLU	64	0.56 (0.36,0.87)^f^		0.45 (0.28,0.73)^f^	

Significant *P* values are shown in bold.

^a^Based on multivariable Fine and Gray model adjusted for FTBI partner agent for neutrophil engraftment, and FTBI partner agent, HCT period and disease status for platelet engraftment.

^b^Based on multivariable Fine and Gray model adjusted for Ph status and F to M HCT for relapse, and age, HCTCI, disease status, and F to M HCT for NRM

^c^Based on multivariable Cox regression model adjusted for disease status.

^d^Based on multivariable Fine and Gray model adjusted for sex and donor type.

^e^Based on multivariable Fine and Gray model adjusted for donor type, F to M HCT, and HCT period.

^f^Based on multivariable Fine and Gray model adjusted for F to M HCT, and HCT period. Donor type was not included due to collinearity.

### Relapse/NRM

**Fig. 2:**
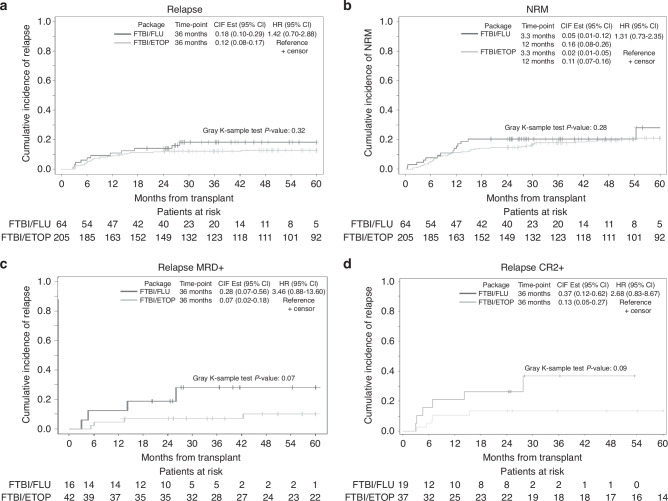
Kaplan Myer curves comparing transplant outcomes after FTBI/FLU and FTBI/ETOP packages. **a** Relapse, **b** Non-relapse mortality, **c** Subgroup analysis of relapse outcomes in patients undergoing HCT with MRD+ status, and **d** Subgroup analysis of patients undergoing HCT at CR2+.

We did not observe any significant effect from either conditioning regimen on relapse at 3 years post-HCT (*P* = 0.32; Fig. 2a). By multivariate analysis, as expected, relapse was significantly higher in patients with Philadelphia-positive status (*p* = 0.02) in all patients and regardless of conditioning regimen, see Table 2. In subgroup analysis, we did not observe a significant difference in relapse between the 2 regimens (FTBI/FLU Vs. FTBI/ETOP) based on Philadelphia chromosome status (Ph+ vs. Ph- vs. Ph-like) (*p* = 0.50 vs. *p* = 0.67 vs. *p* = 0.44, respectively). Administration of either regimen did not impact NRM at 100 days and 1-year post-HCT (*p* = 0.28; Fig. 2b). In multivariate analysis, among other risk factors, disease status (CR1 vs. CR2+) and female donor to male recipient had a significant impact on NRM (*p* = 0.02 and *p* = 0.01, respectively; Table 2).

In patients with available MRD data (*n* = 191/269), patients undergoing HCT with MRD+ disease (*n* = 58/191) had a trend towards higher risk of relapse if they underwent FLU package compared to ETOP package (*p* = 0.07; Fig. 2c), while conditioning regimen/donor package had no impact on relapse risk in patients undergoing HCT with MRD- disease (*n* = 133/191).

In subgroup analysis of patients undergoing HCT in CR1 (*n* = 213/269), relapse risk was not impacted by the conditioning regimen/donor package (*p* = 0.84); and *p* value remained insignificant regardless of MRD status (*p* = 0.91 in MRD+ and *p* = 0.73 in MRD- subgroups, data not shown). However, patients undergoing HCT in CR2 or higher had a trend towards higher risk of relapse when undergoing FLU package compared to ETOP package (*p* = 0.09; Fig. 2d). In patients who underwent HCT at CR2 and MRD- status (*n* = 25), donor-conditioning package did not significantly impact relapse at 3-years post-HCT (*P* = 0.45). While the number of events is too small to draw a definitive conclusion, among patients who underwent HCT in CR2 with MRD+ status, three out of four who received FTBI-FLU relapsed, whereas none of the patients relapsed among the eight who received FTBI-ETOP.

### Survival outcomes

**Fig. 3:**
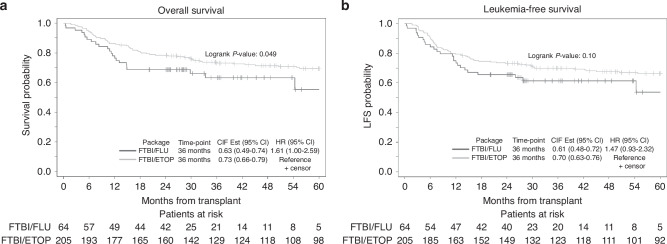
Kaplan Myer curves comparing transplant outcomes after FTBI/FLU and FTBI/ETOP packages. **a** Overall survival, **b** Leukemia-free survival.

At 3 years post-HCT, OS was significantly higher in FTBI/ETOP group (*p* = 0.049; Fig. 3a). However, the significant effect of conditioning regimen on OS was lost by MVA (*p* = 0.07; Table 2). Among other risk factors, disease status (CR1 vs. CR2+) had a significant impact on OS (HR = 2.34; 95% CI: 1.49–3.67, *p* < 0.001). In subgroup analysis, based on disease status at the time of HCT (CR1 or CR2+), we did not detect any difference in 3-year OS based on conditioning regimen in patients undergoing HCT in CR1. However, there was a trend towards better OS in the FTBI-ETOP group when patients received HCT in CR2+ (28% vs. 59%, *p* = 0.06). In the subgroup analysis of patients with available MRD, we did not observe any significant difference in OS at 3 years post HCT by MRD status (positive vs. negative) and between the two regimens (*p* = 0.43 and *p* = 0.12 for MRD+ and MRD- patients, respectively).

We did not observe any significant effect from either conditioning regimen on LFS (*p* = 0.10; Fig. 3b); however, disease stats (CR1 vs CR2+) significantly impacted LFS by both UVA and MVA (*p* < 0.001 and *p* = 0.001, respectively; Table 2). Also, we detected a trend in 3-year LFS in the CR2+ subgroup that received FTBI/ETOP compared to FTBI/FLU (32% vs. 56%, *p* = 0.08). MRD status did not impact the LFS at 3 years post-HCT (*p* = 0.28 for both MRD-positive and negative status).

Lastly, the composite endpoint of GRFS at 1-year was not significantly different among patients receiving FTBI/FLU [0.48 (95% CI: 0.35–0.59)] and FTBI/ETOP [0.47 (95% CI: 0.40–0.53)], (*p* = 0.80). No other risk factors were shown to impact GRFS significantly by UVA or MVA (Supplementary Table 1). One-year GRFS was not impacted by patients’ CR status (CR1 or CD2+) or MRD status (positive or negative) in a subgroup analysis and based on the conditioning regimen (*p* = 0.65 for CR1, 0.10 for CR2+, 0.72 for MRD+ and 0.40 for MRD−).

### GVHD outcomes

**Fig. 4:**
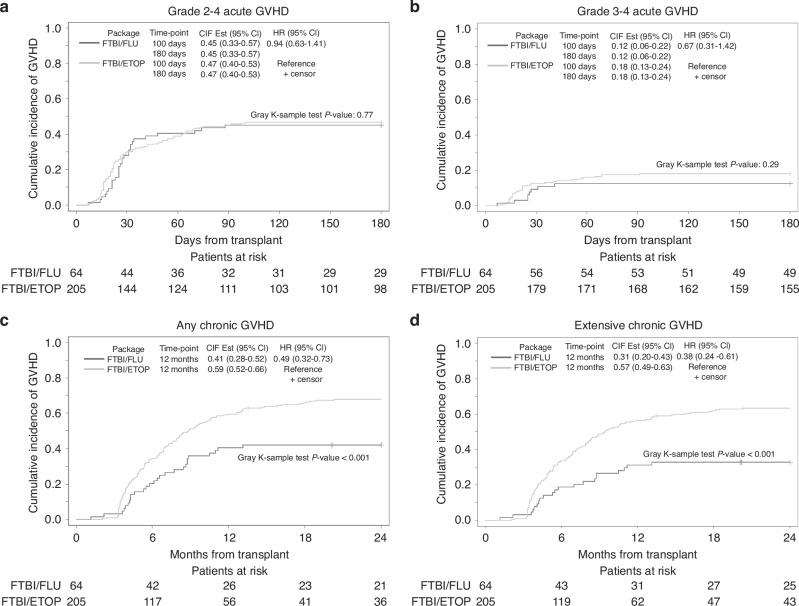
Graft-versus host disease outcomes after FTBI/FLU and FTBI/ETOP packages. **a** Grade 2–4 acute GVHD at days 100 and 180 post-transplant, **b** grade 3–4 acute GVHD at days 100 and 180 post-transplant, **c** Any chronic GVHD at 12 months after transplant, and **d** Extensive chronic GVHD at 12 months after transplant.

Grade 2–4 and 3–4 acute GVHD at 100 days were not significantly different between the two groups (Fig. 4a, b). By MVA, sex (male vs female) significantly impacted grade 2–4 and grade 3–4 acute GVHD and donor type (matched vs MMUD vs haplo) significantly impacted only grade 2–4 GVHD (Table 2). Subgroup analysis based on CR status or MRD status did not impact acute GVHD outcomes (data not shown).

At 1-year post-HCT, the cumulative incidence of any chronic GVHD and extensive chronic GVHD was significantly higher in ETOP patients by both UVA *p* < 0.001 (Fig. 4c, d) and MVA (*p* = 0.01 for any chronic GVHD and *p* = 0.001 for extensive chronic GVHD, Table 2). Other risk factors impacting any chronic GVHD by MVA were female donor to male recipient (*p* = 0.01), and HCT period (*p* = 0.01). Other risk factors impacting severe chronic GVHD by MVA were donor type (*p* = 0.02), female donor to male recipient (*p* = 0.01), and HCT period (*p* = 0.001), (Table 2). Lastly, chronic GVHD outcomes (both any and extensive) were significantly impacted by conditioning regimen in patients undergoing HCT in CR1, CR2+, MRD+ or MRD-negative status (data not shown).

### Cause of death (COD)

There was a total of 84 deaths in both groups (30% in ETOP and 34% in FLU). The most common COD were infection (32% Vs. 36%, *p* = 0.69), chronic GVHD (29% Vs. 13%, *p* = 0.15), relapse (21% Vs. 27%, *p* = 0.55), and organ dysfunction (10% vs. 13%, *p* = 0.59) in ETOP and FLU groups, respectively. COD as unknown was reported in seven patients (five and two patients in ETOP and Flu groups, respectively).

## Discussion

The optimal FTBI-based myeloablative conditioning regimen for adults with ALL undergoing alternative donor HCT with PTCy has remained undetermined due to the lack of prospective, randomized trials. Administration of 12 to 13.2 Gy of FTBI-based myeloablative conditioning regimens in fit and young adult patients has shown superior survival outcomes when compared with non-radiation-based regimens, due to stronger anti-leukemic effects [5, 27–30] and less toxicity from FTBI-based regimens [31]. TBI-based regimens are frequently used with cyclophosphamide (Cy) or ETOP in the matched donor setting [32]. However, in the alternative-HCT setting, and with use of PTCy as GVHD prophylaxis, combining radiation with Cy or ETOP would presumably cause significant toxicity. Therefore, radiation in combination with FLU, with less overlapping toxicity, could be more beneficial [33, 34]. Here, we retrospectively compared HCT outcomes in patients with ALL undergoing FTBI-based conditioning with FLU (matched donor) or ETOP (HAPLO and mismatched donor).

In our analyses, outcomes of patients with ALL undergoing HCT from a mismatched (related or unrelated) donor and receiving the FLU package compared favorably with those undergoing match donor-HCT and receiving the ETOP package. Three-year OS, LFS, and relapse were not significantly different among patients receiving either package. Engraftment and GVHD outcomes in patients of the FLU-package is in accordance with other studies using PTCy as GVHD prophylaxis [19, 20], where engraftment is delayed and cumulative incidence and severity of chronic GVHD is less. As expected, while neutrophile and platelet engraftment were delayed with the use of the PTCy, the overall cumulative incidence of acute GVHD (grade 2–4 and 3–4) were similar among patients receiving either package. Of note, chronic GVHD (any and extensive) were significantly higher in the ETOP-package patients (*p* = 0.01 and *p* = 0.001, respectively), which may be explained by the preventive effect of PTCy through removing activated alloreactive donor and recipient T cell clones at early days after HCT, while maintaining the regulatory T cells [35–37].

Altogether, these outcomes led to comparable 1-year GRFS of 48% for the FLU-package and 47% for the ETOP-package, (*p* = 0.8). Although short-term GRFS was similar, we expect that mortality due to chronic GVHD complications and/or relapse will affect long-term outcomes. COD by conditioning regimen at the time of this report, showed a more than 2 times increased death from chronic GVHD with ETOP compared with FLU (29% vs. 13%), but long-term follow-up is needed to confirm these results.

Our HCT outcomes when comparing Flu and ETOP package were similar (OS/LFS/relapse at 3 years being 63%, 61%, and 18% Vs. 73%, 70%, and 12% and GRFS at 1 year of 47%, and 46%, respectively) to what has been published in a small series (27 patients with ALL) in the HAPLO setting after TBI (12 Gy)+ Flu regimen with 4-year OS of 62%, LFS and relapse rate of 51% and 35%, respectively [38]; as well as a larger registry study with the addition of MMUD (3-years OS and relapse rate of 44% and 37% in HAPLO and 1-year OS of 87% in MMUD) [19, 20].

Historically, patients with ALL treated with FTBI-based therapy in CR2 have higher relapse risk compared to those transplanted in CR1 [39, 40]. In our analysis, after adjusting for disease status (CR1 vs. CR2+), there was no significant difference in the relapse risk and OS when comparing FLU and ETOP packages. However, when we restricted our analysis to patients in CR2, ETOP appeared to be superior in disease control and survival compared to FLU (*p* = 0.06 for OS, *p* = 0.08 for LFS).

MRD is one of the impactful factors in HCT outcome resulting in inferior LFS and OS [41–43]. In our analysis, a total of 65% of patients had their pre-HCT MRD outcomes and 30% of total were MRD+. Relapse risk was higher in MRD+ patients when treated with FLU compared to ETOP (28% vs 7%, *P* = 0.071) while relapse rate was comparable between the two packages in MRD-negative patients.

In our analyses, relapse was significantly higher in the Ph-like group in all patients (*p* = 0.02). However, in a subgroup analysis we did not observe any difference in relapse between the two regimens based on Ph chromosome status (*p* = 0.43). Our OS, LFS, and relapse outcomes are aligned with our previously published data of 3.5-year outcomes of HCT in adults with non-Ph-like ALL compared to Ph-like ALL who underwent HCT with all types of donors and conditioned with either reduced intensity or myeloablative conditioning regimens [44]. Dissecting the effect of FTBI-based conditioning regimens on HCT outcomes, and especially disease relapse in Ph-positive ALL is difficult due to the widespread use of tyrosine kinase inhibitors (TKIs), both preemptively before HCT and/or prophylactically post-HCT, as the TKIs can reduce the risk of relapse independent of the pre-HCT conditioning regimen in this patient population [45].

Despite the strengths of this homogenous retrospective study in ALL patients, our study is limited by its observational/retrospective nature of a single center analysis with low number of patients and the incomplete pre-HCT MRD assessment. The difference in FTBI dose between the two groups (1200 vs 1320 cGy) may have affected the cumulative incidence of relapse and possibly GVHD rates, considering the tissue injury/damage, which could have introduced a bias. Lower doses of fTBI for patients with MRD-negative status may be beneficial and should be studied.

In conclusion, FTBI/FLU with alternative donor and PTCy-based GVHD prophylaxis as a “package” has offered comparable and nontoxic option to FTBI/ETOP, for patients with ALL who are eligible for myeloablative HCT and lack a fully matched donor. Disease status and depth of remission at the time of HCT remained independent predictors for better HCT outcomes in our study regardless of the type of “package” received.

## Supplementary information

Supplementary Table 1

## Data Availability

The datasets generated during and/or analyzed during the current study are available from the corresponding author upon reasonable request.
