# Supplementary material for: Comparing transplant outcomes in ALL patients after myeloablative conditioning in mismatch-related or unrelated donor settings
Source: Bone Marrow Transplant. 2024 Aug 15;59(11):1542–51. doi: 10.1038/s41409-024-02378-0 (PMC11530400; doi:10.1038/s41409-024-02378-0)
Supplement: Supplementary file 1 — Supplementary Table 1 [file 41409_2024_2378_MOESM1_ESM.docx]

| *Supplementary Table 1. GRFS outcomes* | | | | | | | |
| --- | --- | --- | --- | --- | --- | --- | --- |
|  |  | *N* | *1 Yr (95%CI*)* | *HR (95%CI)** | *P** | *Adjusted HR (95%CI)†* | *Wald test P†* |
| Age, years | ≤40 | 157 | 0.471(0.391,0.547) | Reference | 0.95 | Reference | 0.82 |
|  | >40 | 112 | 0.464(0.370,0.553) | 1.01(0.75,1.37) |  | 1.04(0.76,1.40) |  |
| Sex | M | 160 | 0.472(0.392,0.547) | Reference | 0.59 | Reference | 0.63 |
|  | F | 109 | 0.463(0.367,0.553) | 0.92(0.68,1.25) |  | 0.93(0.68,1.26) |  |
| KPS | ≥80 | 251 | 0.458(0.395,0.518) | Reference | 0.29 | Reference | 0.20 |
|  | ≤70 | 18 | 0.611(0.353,0.792) | 0.71(0.37,1.34) |  | 0.65(0.34,1.25) |  |
| HCTCI | 0 | 84 | 0.476(0.366,0.578) | Reference | 0.75 | Reference | 0.81 |
|  | 1-2 | 98 | 0.495(0.392,0.589) | 1.00(0.69,1.44) |  | 1.01(0.70,1.46) |  |
|  | ≥3 | 87 | 0.430(0.325,0.531) | 1.13(0.77,1.64) |  | 1.12(0.77,1.63) |  |
| Ph status | + | 102 | 0.515(0.414,0.607) | Reference | 0.080 | Reference | 0.100 |
|  | - | 138 | 0.464(0.379,0.544) | 1.19(0.86,1.65) |  | 1.14(0.81,1.59) |  |
|  | Phlike | 29 | 0.321(0.161,0.493) | 1.72(1.06,2.79) |  | 1.70(1.05,2.75) |  |
| Poor risk | No | 66 | 0.470(0.346,0.584) | Reference | 0.91 | Reference | 0.90 |
|  | Yes | 203 | 0.468(0.397,0.535) | 0.98(0.69,1.38) |  | 1.02(0.72,1.45) |  |
| ALL type | B-ALL | 233 | 0.468(0.402,0.530) | Reference | 0.89 | Reference | 0.84 |
|  | T-ALL | 36 | 0.472(0.305,0.623) | 0.97(0.63,1.51) |  | 0.95(0.61,1.48) |  |
| Disease status | CR1 | 213 | 0.488(0.419,0.554) | Reference | 0.098 | Reference | 0.100 |
|  | CR2+ | 56 | 0.393(0.266,0.517) | 1.34(0.94,1.92) |  | 1.35(0.94,1.92) |  |
| Donor type | Matched | 196 | 0.474(0.403,0.542) | Reference | 0.84 | Reference | 0.94 |
|  | MMUD | 26 | 0.480(0.278,0.656) | 1.16(0.70,1.90) |  | 1.09(0.66,1.81) |  |
|  | HAPLO | 47 | 0.435(0.290,0.571) | 1.03(0.69,1.54) |  | 1.02(0.68,1.52) |  |
| F Donor to M | No | 206 | 0.459(0.389,0.525) | Reference | 0.59 | Reference | 0.54 |
|  | Yes | 63 | 0.500(0.371,0.616) | 0.91(0.63,1.30) |  | 0.89(0.62,1.28) |  |
| HCT period | 2010-2014 | 83 | 0.482(0.371,0.584) | Reference | 0.78 | Reference | 0.90 |
|  | 2015-2020 | 186 | 0.462(0.389,0.532) | 1.05(0.75,1.45) |  | 1.02(0.73,1.42) |  |
| FTBI+ | VP-16 | 205 | 0.466(0.396,0.532) | Reference | 0.80 | Reference | 0.68 |
|  | FLUDARABINE | 64 | 0.476(0.349,0.593) | 0.96(0.67,1.37) |  | 0.93(0.65,1.33) |  |

* Based on Kaplan-Meier curve, univariate analysis, and log-rank test.

*†* Based on multivariable Cox regression model adjusted for disease status
